# Phase II evaluation of sunitinib in the treatment of recurrent or refractory high‐grade glioma or ependymoma in children: a children's Oncology Group Study ACNS1021

**DOI:** 10.1002/cam4.713

**Published:** 2016-04-25

**Authors:** Cynthia Wetmore, Vinay M. Daryani, Catherine A. Billups, James M. Boyett, Sarah Leary, Rachel Tanos, Kelly C. Goldsmith, Clinton F. Stewart, Susan M. Blaney, Amar Gajjar

**Affiliations:** ^1^ Department of PediatricsAflac Cancer and Blood Disorders CenterChildren's Healthcare of Atlanta and Emory University School of Medicine1760 Haygood Drive NEAtlantaGeorgia30322; ^2^ Department of Pharmaceutical SciencesSt. Jude Children's Research HospitalMemphisTennessee38105; ^3^ Department of BiostatisticsSt. Jude Children's Research HospitalMemphisTennessee38105; ^4^ Department of Hematology/OncologySeattle Children's HospitalSeattleWashington98145; ^5^ Pediatric OncologyClinical Care CenterBaylor College of MedicineHoustonTexas77030; ^6^ Department of OncologySt. Jude Children's Research HospitalMemphisTennessee38105

**Keywords:** Ependymoma, Pediatric, recurrent brain tumor, tyrosine kinase inhibitor

## Abstract

Sunitinib malate is a small multi‐targeted tyrosine kinase inhibitor that inhibits vascular endothelial growth factor receptor (VEGFR), platelet‐derived growth factor receptor (PDGFR) and stem cell factor receptor (KIT), which are highly expressed by some high‐grade brain tumors. We conducted a phase II study to estimate the efficacy and further characterize the pharmacokinetics of sunitinib in pediatric patients with recurrent or refractory high‐grade glioma (Stratum A) or ependymoma (Stratum B). This was a prospective, multicenter Phase II trial conducted through the Children's Oncology Group (ClinicalTrials.gov Identifier NCT01462695). Sunitinib, 15 mg/m2, was orally administered once daily for 4 weeks every 6 weeks. The safety and tolerability of sunitinib, an estimate of progression‐free survival (PFS), analyses of sunitinib pharmacokinetics (PK) and pharmacodynamics modulation of plasma VEGF and VEGFR2 were also assessed. Thirty eligible patients (17 patients on Stratum A, 13 patients on Stratum B) were enrolled and 29 patients were evaluable for response. Sunitinib was reasonably well tolerated in children with recurrent ependymoma or high‐grade glioma. Most adverse events were of mild‐to‐moderate severity and manageable with supportive treatment. While there was a statistically significant modulation of plasma VEGFR2 with sunitinib exposure, there were no sustained tumor responses. Both strata were closed at time of planned interim analysis as there was not sufficient efficacy associated with sunitinib in children with recurrent brain tumors. Sunitinib was well tolerated in children and young adults with recurrent high‐grade glioma or ependymoma but had no single agent objective antitumor activity in these patients.

## Introduction

Children with high‐grade gliomas (HGG) or ependymomas have a poor prognosis, despite advances in surgery, radiation therapy and chemotherapy. For children with recurrent or refractory disease, a standard therapy does not exist and participation on a clinical trial may offer the best chance of an effective treatment. Amplification and activation of several receptor tyrosine kinases (RTKs) including epidermal growth factor receptor, platelet‐derived growth factor receptors (PDGFR), stem cell factor receptor (KIT), and vascular endothelial growth factor receptors (VEGFR), are among the most common molecular changes characterizing high‐grade gliomas in adults and are found in approximately 30% of pediatric patients 1, 2, 3. VEGFR, PDGFR and their respective ligands are highly expressed in ependymoma and glioblastoma cell lines as well as in primary tumor tissues 1, 2.

The growth of solid tumors is generally limited by their vascular supply and, thus, inhibitors of angiogenesis have become attractive new targets for cancer therapy 4. Sunitinib malate (SU011248) is an orally bioavailable receptor tyrosine kinase inhibitor that antagonizes cellular signaling of multiple targets involved in tumor proliferation and angiogenesis, with specific activity against VEGFR, PDGFR, KIT, and Fms‐like tyrosine kinase 3 receptor 5. Sunitinib has FDA approval in the United States for use in adults with metastatic/unresectable gastrointestinal stromal tumors (GIST) and renal cell carcinoma. Sunitinib has been well‐tolerated in early clinical studies in children 6 and adults 7 with GIST, and in adults with metastatic solid tumors 8, 9.

Given the redundancy of several molecular pathways simultaneously involved in tumorigenesis of ependymoma and high‐grade glioma, we reasoned that the multi‐targeted specificity of sunitinib was an appealing strategy for this study. Based upon this rationale and results of the pediatric Phase I study 6, we conducted a Phase II study of once daily dosing of sunitinib in children and young adults with recurrent or refractory ependymoma or HGG, excluding diffuse intrinsic pontine glioma.

## Subjects and Methods

### Study design and treatment

This was a Phase II, open label clinical trial of sunitinib in children and young adults with recurrent or progressive HGG or ependymoma (ClinicalTrials.gov Identifier: NCT01462695). The trial was approved by the Institutional Review Boards at all participating centers as well as the National Cancer Institute Pediatric Central IRB. All subjects or their parent/legal guardian signed a document of informed consent and assent was obtained as appropriate.

Participants were enrolled on Stratum A (recurrent/progressive/refractory HGG) or Stratum B (recurrent/progressive/refractory ependymoma) based on their initial histopathological diagnosis. The primary objective of the study was to estimate the overall response rate (ORR); defined as the percentage of patients who experienced a confirmed complete (CR) or partial response (PR) that was sustained for at least 8 weeks. Secondary endpoints included: assessment of the safety and tolerability of sunitinib in children or young adults who had not received prior anthracycline or radiotherapy involving the heart, estimation of the progression‐free survival (PFS), evaluation of sunitinib pharmacokinetic disposition, and exploration of potential changes in plasma levels VEGF and VEGFR2 levels 10.

Based on the recommended phase II dose from the pediatric phase I COG protocol, ADVL0612, sunitinib was administered at a dosage of 15 mg/m^2^/dose in 6 week cycles. Each cycle was comprised of once daily oral sunitinib for 28 days followed by a 14‐day rest period. Patients were monitored for toxicity and had their first disease status evaluation after two cycles (12 weeks) of therapy. At that time if subjects had progressive disease, they were removed from protocol therapy. If a subject was deemed by their local provider to have a complete response, partial response or stable disease, they could continue on sunitinib therapy and were required to have radiographic confirmation of objective response 8 weeks after the initial response assessment. Tumor response was determined by the changes in size with use of the maximal 2‐dimensional cross‐sectional tumor measurements, using either T1‐ or T2‐weighted images. The tumor response definitions used were as follows: CR disappearance of all target lesions; PR, ≥50% decrease in size of all target lesions; progressive disease (PD), ≥25% increase in the size of any target lesion or the appearance of new lesions; and stable disease (SD), neither sufficient decrease nor increase in tumor size to qualify for PR or PD, respectively 11.

### Eligibility criteria

Patients between the ages of 18 months and 22 years with a performance status corresponding to ECOG scores of 0, 1 or 2 who had histological confirmation of HGG or ependymoma with unequivocal evidence of disease progression that was measurable were eligible. Patients could have received no more than two prior treatment regimens. Intervals from prior therapy to enrollment included at least 2 weeks from prior focal (salvage) radiation therapy; 24 weeks from prior full field radiotherapy; and 3 to 6 weeks from previous myelosuppressive antitumor therapy, depending upon the specific agent and count recovery. Patients had to be capable of taking oral medication and could not have had prior therapy with sunitinib or with another targeted inhibitor of VEGF, PDGFR or KIT pathways. Patients who were receiving dexamethasone had to be on a stable or decreasing dose for at least 7 days prior to enrollment. All participants were required to have adequate bone marrow and organ function. Exclusion criteria included use of enzyme‐inducing antiepileptic medications within 7 days prior to enrollment; prior radiation therapy that included the mediastinal region; prior therapy with known risk for cardiovascular complications (e.g., anthracycline therapy); history of a cerebrovascular accident, recent hemorrhage or other significant thromboembolic event within 12 months prior to enrollment.

### Assessments

Baseline evaluations included medical history, physical examination, tumor imaging with magnetic resonance imaging (MRI), laboratory evaluation (hematology, urinalysis, blood chemistry, liver function studies, blood pressure and pregnancy tests), and electrocardiogram (ECG). Hematology and blood chemistries were performed prior to each cycle. ECG and MRI evaluations were performed before each odd‐numbered treatment cycle. The response assessment (MRI) was performed at the end of the dosing in cycle 2, followed by every 2 cycles as previously described 11. Objective responses were required to be sustained for at least 8 weeks after the initial CR/PR determination. Toxicity was graded according to the National Cancer Institute Common Toxicity Criteria version 4.0.

### Treatment modifications

Sunitinib dose modification was required if patients developed a nonhematological toxicity that was: grade 4; grade 3 with standard exclusions for hepatic toxicity (Grade 3 elevation of ALT, AST and/or Bilirubin that resolved to ≤ Grade 2 within 7 days of study drug interruption and did not recur), electrolyte abnormalities, non‐neutropenic fever or infection, or grade 2 that persisted for ≥7 days and intolerable or medically significant. Dose modifications were also required for grade 4 neutropenia or thrombocytopenia. Specific dose modifications criteria were provided for hepatic toxicity, hand‐foot syndrome, QTc prolongation, flu‐like symptoms, and hypertension.

### Pharmacokinetics and pharmacodynamics

For pharmacokinetic analysis, single steady‐state trough samples were obtained from all consenting patients immediately prior to dose administration during cycle 1 on days 7, 14, 21, and 28 along with cycle 2 on days 1 and 28. In addition, optional serial pharmacokinetic studies on cycle 1 day 1 were acquired at 2, 4, 6–8, and 24 (±1) h postdose. Plasma concentrations of sunitinib and its primary active metabolite, SU012662, were measured by liquid chromatography/tandem mass spectrometry (BASi, West Lafayette, IN) as previously described 12. Sunitinib and SU012662 plasma concentration‐time data were modeled by a one compartment model with first‐order absorption and lag time using the maximum likelihood function in ADAPT V 13. Pharmacokinetic parameters estimated included apparent oral clearance (CL/F) of sunitinib and metabolite, apparent volume of distribution (V/F) of sunitinib and metabolite, absorption rate constant (K_a_), and the absorption lag time (t_lag_). Area under the plasma concentration‐time curve from zero to the last measurable time point (AUC_0‐Tlast_) was calculated using the log linear trapezoidal rule for both sunitinib and SU012662.

Peripheral blood was collected into EDTA‐containing tubes from consenting patients at baseline (prior to starting sunitinib), and at days 14 and 28 of course one of sunitinib therapy. Plasma was separated by centrifugation, and frozen at −80°C. Plasma samples were then batched, prepared and each of the soluble proteins (VEGF and VEGFR2) was detected by enzyme‐linked immunosorbant assay (ELISA) according to the manufacturer's protocol (R&D Systems, Minneapolis, MN). Exact Wilcoxon signed‐rank tests were used to compare differences in VEGF and VEGFR2 levels from baseline. The course one, day 1 level was considered the baseline.

### Statistical considerations

The statistical design for the primary objective was based on Simon's minimax two‐stage design. The statistical design had 90% power for a true response rate of 30% in stratum A and 25% in stratum B. The Type I and II error rates for both strata were set at 10%. The trial was structured to have the sample size of 25 for stratum A and 20 for stratum B. If one or fewer patients were observed among the first stage to have a response, the stratum was closed at interim evaluation due to lack of sufficient efficacy. Sunitinib was deemed not worthy of further investigation if the true response rate was less than 10% in stratum A and 5% in stratum B. Interim analysis was done after enrollment of 16 patients on Stratum A and 13 on Stratum B. Any eligible patient who received any sunitinib was considered evaluable for the primary objective.

Progression‐free survival was defined as the time interval from date of study enrollment to date of first event (relapsed or progressive disease or death from any cause) or to date of last contact for patients without events. Survival was defined as the time interval from study enrollment to date of death from any cause, or to date of last contact for patients who had not died. Progression‐free and overall survival distributions were estimated, using the method of Kaplan and Meier. Frozen data as of September 30, 2014 were used for this analysis.

## Results

### Patient characteristics

Thirty patients were enrolled in the study between January 2012 and June 2013; seventeen on stratum A and thirteen on stratum B. All patients were deemed eligible for the protocol, though 1 stratum A patient did not receive any sunitinib and was not evaluable for the primary objective of estimating the sustained response rate. Patient characteristics for the 29 evaluable patients are presented in Table 1 and the median number of cycles received was two (range, 1–5). All patients had previously received treatment that included radiation therapy (for patients >3 years of age) and/or chemotherapy at time of initial diagnosis.

**Table 1:** Patient characteristics for ACNS1021 patients who received study drug (*n *= 29)

	Stratum A Recurrent HGG (*n *= 16)	Stratum B Recurrent Ependymoma (*n *= 13)	All Patients Who Received Study Drug (*n *= 29)
Age at study enrollment (years)			
Median	14.5	12.0	13.4
Range	4.7–19.9	3.0–16.9	3.0–19.9
Sex			
Male	12	6	18
Female	4	7	11
Race			
White people	13	11	24
Black people	3	0	3
Other	0	2	2
ECOG (Zubrod) performance score^1^	7 (44%)7 (44%)2 (12%)	10 (77%)2 (15%)1 (8%)	17 (59%)9 (31%)3 (10%)
Patient's registry stage			
Local	10	10	20
Regional	1	1	2
Distant	1	1	2
Unknown	1	1	2
Not applicable	3	0	3
Tumor grade^2^			
WHO Grade 2		3	
WHO Grade 3	9	5	
WHO Grade 4	7		
Number of patients who had prior RT	15	10	25
Number of patients who had prior CT	14	8	22
Number of sunitinib cycles received			
Median	1	2	2
Range	1–2	1–5	1–5

0: Fully active able to carry on all predisease performance without restriction.

1: Restricted in physically strenuous activity but ambulatory and able to carry out work of a light or sedentary nature, for example, light house/office work

2: Ambulatory and capable of all self‐care but unable to carry out any work activities. Up and about more than 50% of waking hours.

HGG: high‐grade gliomas; RT: radiation therapy; CT: chemotherapy.

^1^ECOG performance score definitions. ^2^Tissue was not available for central pathology review for five of the patients.

### Sunitinib exposure

Among the 29 patients who received at least one dose, the median dose of sunitinib received during reporting period 1 *(*cycles 1 and 2*)* was 700 mg (range, 143.75 mg – 1750 mg). The patient with lowest exposure (143.75 mg) received 7 days at original dose of 12.5 mg, then the drug was held 12 days due to grade 3 elevation of amylase, and the patient then resumed sunitinib therapy at a reduced dose (6.25 mg) for an additional 9 days before he was taken off therapy for progressive disease. Two patients on Stratum B continued on sunitinib therapy for >2 cycles but neither had a sustained response.

### Response and survival

No sustained radiographic responses were observed in either arm and the study was closed at time of interim analysis due to lack of sustained objective response (PR or CR ≥8 weeks). Table S1 summarizes the primary reasons patients came off study treatment.

The observed response rate for stratum A was 0% (95% Blyth‐Still‐Casella confidence interval [CI], 0%–19.8%). Three stratum A patients had stable disease at the first reporting period evaluation and then came off treatment (2 due to “physician determines it is in the patient's best interest” and 1 for “occurrence of a new or worsening hemorrhage on brain MRI”). Four additional stratum A patients were not evaluated for disease status at the first reporting period and then came off protocol treatment (2 due to “physician determines it is in the patient's best interest,” 1 due to “death” and 1 due to “refusal of further protocol therapy by patient/parent/guardian”). These patients were considered as failures in the primary analysis of overall response.

The observed response rate for stratum B was also 0% (0%–22.5%). Although one stratum B patient was noted to have a partial response after two cycles, the response was not sustained for a minimum of eight weeks. An additional Stratum B patient had stable disease after two cycles but this was not sustained. The remaining 11 stratum B patients had PD on their initial response assessment.

**Figure 1:**
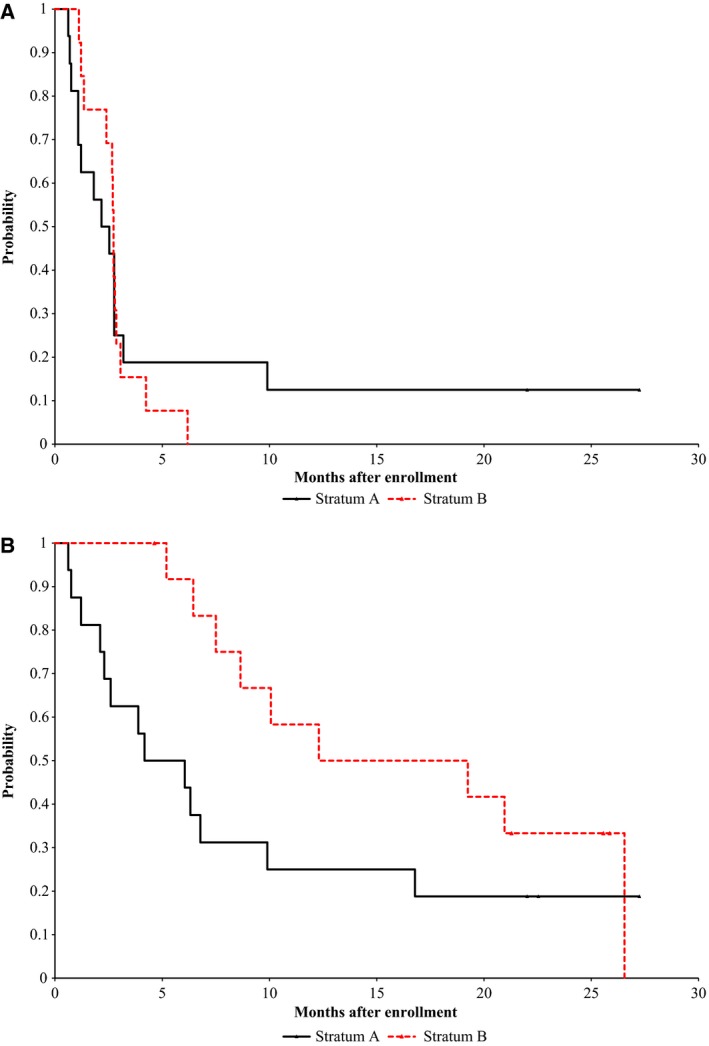
Progression free (A) and Overall (B) survival.

Of the 16 evaluable patients on stratum A, the median time to progression was 72 days (95% CI, 33–84), and of the 13 evaluable patients on Stratum B, the median time to progression was 83 days (95% CI, 41–87) (Fig. 1A). OS for patients in both strata is shown in Figure 1B.

### Toxicity

Sunitinib was generally well tolerated with toxicity typically infrequent and mild. Adverse events for cycles 1 and 2 are shown in Table 2. Grade 3–4 AEs that were deemed at least possibly related to sunitinib occurred in 11 patients and included decreased lymphocytes (*n* = 1), decreased neutrophils (*n* = 7), transaminase elevation (*n* = 1) and serum amylase or lipase elevation (*n* = 1). Only 1 patient experienced the typical maculo‐papular skin rash associated with sunitinib. No other sunitinib‐related adverse events were reported during sunitinib therapy after cycle 2 or in the follow‐up period. Cumulative sunitinib toxicity could not be assessed as most patients came off protocol therapy after the first reporting period.

**Table 2:** Toxicities during reporting period 1 (cycles 1 and 2) by stratum (adverse events *at least possibly related* to study drug**).**

Toxicity	Stratum A HGG *n* = 16				Stratum B Ependymoma *n* = 13			
	Grade				Grade			
	1	2	3	4	1	2	3	4
	*n*	*n*	*n*	*n*	*n*	*n*	*n*	*n*
Alanine aminotransferase increased			1					
Aspartate aminotransferase increased			1					
Lipase increased			1					
Lymphocyte count decreased								1
Neutrophil count decreased			2				4	1
Serum amylase increased			1					
White blood cell decreased							1	
Diarrhea							1	
Fatigue			1					
Intracranial hemorrhage	1			1			1	
Rash maculo‐papular		1						
Skin and subcutaneous tissue disorders ‐ Other (rash, acne)			1					

### Pharmacokinetic and pharmacodynamics

**Figure 2:**
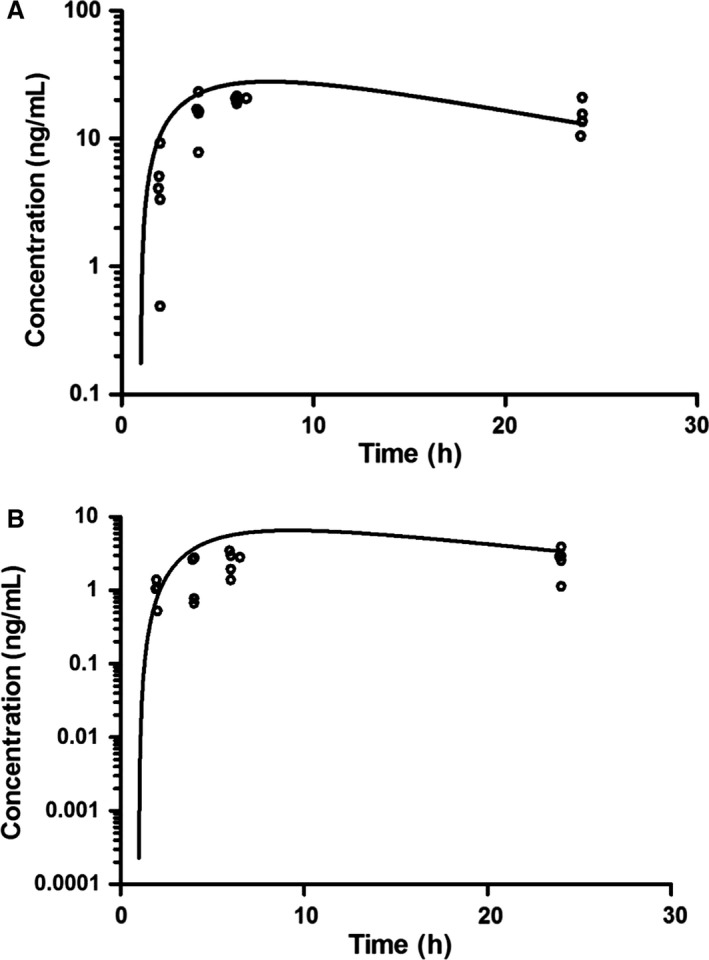
(A) Sunitinib plasma concentration‐time profile; (o) represents observed concentrations and best fit line from the average parameter estimates of five patients on Cycle 1 Day 1. (B) SU012662 plasma concentration‐time profile; (o) represents observed concentrations and the line represents the average parameter estimates of 5 patients on Cycle 1 Day 1.

Predose steady‐state trough plasma samples were collected for pharmacokinetic analysis during cycles 1 and 2 from 23 patients. A summary of the sunitinib steady‐state concentrations is shown in Table 3. Five patients consented to serial plasma pharmacokinetic studies on cycle 1 day 1 and the concentration‐time profiles for sunitinib and its metabolite, SU012662, are shown in Figures 2A and 2B, respectively. The median (18.4–23.2 ng/mL) sunitinib maximum concentration and time to maximum concentration were 21.8 ng/mL and 8.6 h, respectively. The median (2.2–5.1 ng/mL) SU012662 maximum concentration and time to maximum concentration were 3.8 ng/mL and 11.5 h. (8.3–12.5 h), respectively. The pharmacokinetic parameters of sunitinib and SU012662 for cycle 1 day 1 are summarized in Table S2.

**Table 3:** Sunitinib and Total (Sunitinib + SU012662) steady‐state plasma trough concentrations

		C1D7	C1D14	C1D28	C2D1	C2D28
	*n* =	21	20	20	15	11
Sunitinib (ng/mL)						
	Median	35.5	40.1	37.7	0.24	37.1
	Min	14.6	9.8	14.3	0.1	11.3
	Max	77.2	75.5	77.9	0.96	66.6
Total (Sunitinib + SU012662) (ng/mL)						
	Median	46	55.6	55.4	0.6	58
	Min	19.1	15.5	22.9	0.2	21.8
	Max	94.4	92.8	109.7	1.53	97.5
						
Median total >50 ng/mL	*n* (%)	9 (43)	13 (65)	12 (60)	0	6 (55)

**Figure 3:**
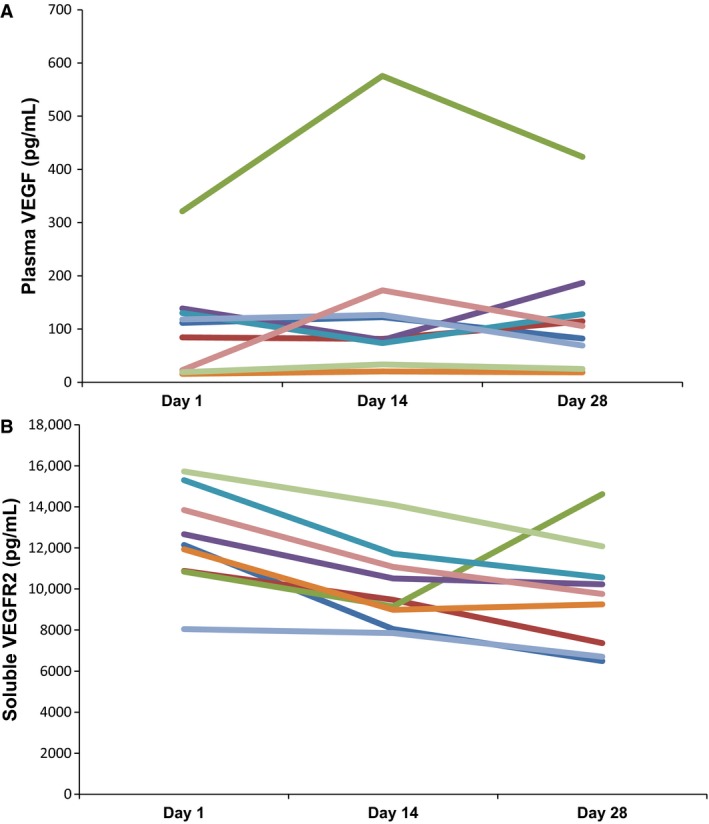
Quantification of plasma VEGF (A) and soluble VEGFR2 (B) in patient plasma prior to sunitinib (Day 1), and 14 and 28 days of continuous sunitinib therapy.

A total of 12 patients consented and had blood samples drawn for PD studies, of these samples only nine resulted in sufficient quality of protein in the plasma for analyses. Figures 3A and 3B show profile plots of VEGF and VEGFR2 levels, respectively. The plasma VEGF level did not change from day 1 to day 14 (*P* = 0.36) or from day 1 to day 28 (*P* = 0.25). However, the VEGFR2 levels decreased in all nine patients after 14 days of exposure to sunitinib (Fig 3B). The median decrease in VEGFR2 from day 1 to day 14 was 2154 pg/mL (range, 197.24–4095). In seven of the nine patients, the VEGFR2 level further decreased from day 14 to day 28 (Fig 3B). There was evidence of a statistically significant change in VEGFR2 level from day 1 to day 14 (*P* = 0.004); and marginal evidence of a significant decrease from day 1 to day 28 (*P* = 0.055).

## Discussion

The recommended Phase II sunitinib dosage is based upon a pediatric Phase I study of 15 mg/m^2^/day, which was the dosage used in this study 14. In this Phase II study of children and young adults with recurrent/refractory HGG or ependymoma, sunitinib therapy did not result in objective responses or prolonged stable disease. This trial did confirm the tolerability of sunitinib at the recommended maximum tolerated dose established in the pediatric phase I trial 14. Sunitinib therapy was generally well tolerated with hematological toxicity being the most common adverse events. Patients who had prior radiation therapy that included the mediastinum were excluded due to the reported cardiotoxicity associated with some tyrosine kinase inhibitors 15. While eight of 17 patients were removed from protocol therapy due to physician or patient preference, this appears not to be directly related to toxicity as there were relatively few target‐related AE noted and there was no correlation between toxicity and pharmacokinetics (PK).

This study represents the first pharmacokinetic report of sunitinib and its metabolite in pediatric patients with progressive HGG or ependymoma. The estimated apparent oral clearance for sunitinib in the present pediatric population was similar to previous estimates from adult (29.9 L/h/m^2^) and pediatric (19.5 L/h/m^2^) studies, but the metabolite, SU012662, was reported to have a much higher clearance in our study at 103.8 L/h/m^2^ compared to a previous adult value of 17.1 L/h/m^2^ and pediatric clearance of 39.1 L/h/m^2^ 14, 16. This difference may be a result of the plasma sampling schedule, which in the present study was designed to characterize the parent compound and was less likely to capture the terminal phase for SU012662. Mendel and colleagues have previously shown in target modulation experiments that the minimum total plasma sunitinib concentration for inhibition of VEGFR and PDGFR*β* was 50–100 ng/mL 17. Examining steady‐state sunitinib plasma trough concentrations from this study (Table 3), the majority of patients likely did not achieve sufficient plasma exposure for inhibition of the target receptor tyrosine kinases. Lastly, sunitinib and its metabolite are substrates for ABCB1 and ABCG2, which will decrease CNS accumulation and concentrations. It is likely that a combination of these factors contributed to the lack of efficacy observed in this study.

While plasma VEGF levels increased in three of five (60%) patients from C1D14 to C1D28, the change was not statistically significant. However, sVEGFR2 levels significantly decreased in all nine patients by C1D14 after exposure to sunitinib (*P* = 0.004), and showed a marginally significant decrease from days 1 to day 28 (*P* = 0.055). This suggests that there was sufficient plasma sunitinib concentration to modulate sVEGFR2 yet this was not correlated with a clinical response. Interestingly, a recent study in adult patients with HGG, used a higher dose of sunitinib (37.5 mg/day) yet there was no modulation of sVEGFR2 and no clinical response. Previous investigators have found a statistically robust correlation in modulation of VEGF and sVEGFR2 patient plasma levels with exposure to sunitinib, and have proposed that such inhibition may be correlated with tumor response in patients with GIST and renal cell carcinoma 10.

There is evidence that antiangiogenic agents, such as sunitinib may exert their antitumor effects in the endothelial compartment, through the “normalization” of tumor vasculature, and contribute to increased tumor response (cell death) to radiation therapy in solid tumors 18, 19. The addition of sunitinib to low‐dose radiation therapy of PDGF‐driven murine glioblastoma, reduced tumor growth but did not result in significant survival benefit over either modality alone 20. However, a Phase Ib study of sunitinib combined with radiation therapy in patients with primary and metastatic tumors of the CNS resulted in acceptable toxicity, with two of 12 patients achieving PR, and nine patients had SD with a median follow up time of 34 months 21.

Unfortunately, our results indicate that at 15 mg/m^2^, sunitinib is not active as a single agent recurrent HGG or ependymoma. Another consideration is that while ~30% of pediatric tumors highly express PDGFR and VEGFR, these pathways may not be critical, single agent mediators of proliferation of HGG and ependymoma cells. Our findings are consistent with other small‐molecule inhibitors of VEGFR and PDGFR in recurrent high‐grade brain tumors with similarly disappointing findings 22, 23, 24. A cohort of 16 adult patient with recurrent glioblastoma treated with daily sunitinib for 4 weeks, followed by a 2‐week break, did not demonstrate antitumor activity that was any greater than that reported with standard cytotoxic therapies 25. Continuous dosing in adult patients with recurrent HGG also failed to demonstrate any clinical benefit of sunitinib at 37.5 mg per day, despite more significant toxicities in the adult cohort 26. While preclinical evidence that VEGFR and PDGFR play a role in tumor cell proliferation and invasion, the negative results of several clinical trials suggest that single agent modulation of these pathways may not be worth pursuing.

In summary, sunitinib is well tolerated in pediatric patients with recurrent/refractory HGG or ependymoma but had insufficient activity to warrant further investigation of this monotherapy regimen in recurrent HGG and ependymoma. However, the role of sunitinib in combination with radiation and/or cytotoxic chemotherapy may be of potential interest to explore in pediatric patients.

### Conflict of Interest

None declared.

## Supporting information


**Table S1**. Primary off treatment reasons by stratum Supplemental Table II. Compartmental Pharmacokinetic Parameters for Sunitinib and SU012662.

**Table S2**. Compartmental Pharmacokinetic Parameters for Sunitinib and SU012662.
