# Supplementary material for: Phase II evaluation of sunitinib in the treatment of recurrent or refractory high‐grade glioma or ependymoma in children: a children's Oncology Group Study ACNS1021
Source: Cancer Med. 2016 Apr 25;5(7):1416–24. doi: 10.1002/cam4.713 (PMC4944867; doi:10.1002/cam4.713)
Supplement: Supplementary file 1 — Table S1. Primary off treatment reasons by stratum Supplemental Table II. Compartmental Pharmacokinetic Parameters for Sunitinib and SU012662. [file CAM4-5-1416-s001.docx]

| **Primary Reason Off Treatment** | **Stratum A (Recurrent high grade glioma)** | **Stratum B (Recurrent ependymoma)** | **All Patients** |
| --- | --- | --- | --- |
| Progressive disease (> 25 % increase in tumor size) | 6 | 12 | **18** |
| Physician deems in the patient's best interest | 6^#^ | 0 | **6**^#^ |
| Refusal of further protocol therapy by patient/parent | 2 | 0 | **2** |
| Death | 2 | 0 | **2** |
| New or worsening hemorrhage on brain MRI | 1 | 1 | **2** |
| **Total** | **18** | **13** | **29** |
