# Supplementary material for: Phase II evaluation of sunitinib in the treatment of recurrent or refractory high‐grade glioma or ependymoma in children: a children's Oncology Group Study ACNS1021
Source: Cancer Med. 2016 Apr 25;5(7):1416–24. doi: 10.1002/cam4.713 (PMC4944867; doi:10.1002/cam4.713)
Supplement: Supplementary file 2 — Table S2. Compartmental Pharmacokinetic Parameters for Sunitinib and SU012662. [file CAM4-5-1416-s002.docx]

|  | Sunitinib |  | SU012662 |
| --- | --- | --- | --- |
| (n = 5 studies) |  | | |
| CL/F (L/hr/m^2^) | 24.4 (12.1 – 26.1) |  | 103.8 (14.7 – 251.4) |
| V/F (L/m^2^) | 390.3 (307.5 – 836.9) |  | 129.2 (21.7 – 157.3) |
| Ka (hr^-1^) | 0.28 (0.11 – 0.74) |  |  |
| t_lag_ (hr) | 1.01 (0.00 – 2.61) |  |  |
| AUC_0-24_ (ng/mL x hr) | 368.1 (344.1 – 424.6) |  | 62.9 (26.7 – 77.6) |
